# More than 20° posterior tilt of the femoral head in undisplaced femoral neck fractures results in a four times higher risk of treatment failure

**DOI:** 10.1007/s00068-021-01673-5

**Published:** 2021-04-26

**Authors:** Jorn Kalsbeek, Ariaan van Walsum, Herbert Roerdink, Inger Schipper

**Affiliations:** 1 Department of Trauma Surgery, Leiden University Medical Centre, Albinusdreef 2, 2333 ZA Leiden, The Netherlands; 2 Department of Trauma Surgery, Medical Spectrum Twente, Koningsplein 1, 7512 KZ Enschede, The Netherlands; 3 Department of Trauma Surgery, Deventer Hospital, Nico Bolkesteinlaan 75, 7416 SE Deventer, The Netherlands

**Keywords:** Femoral neck fracture, Hip fracture, Posterior tilt, Garden type I and II, Dynamic locking blade plate, Garden classification

## Abstract

**Purpose:**

In this study, we aimed to determine the correlation between the preoperative posterior tilt of the femoral head and treatment failure in patients with a Garden type I and II femoral neck fracture (FNF) treated with the dynamic locking blade plate (DLBP).

**Methods:**

Preoperative posterior tilt was measured in a prospective documented cohort of 193 patients with a Garden type I and II FNF treated with the DLBP. The correlation between preoperative posterior tilt and failure, defined as revision surgery because of avascular necrosis, non-union, or cut-out, was analyzed.

**Results:**

Patients with failed fracture treatment (5.5%) had a higher degree of posterior tilt on the initial radiograph than the patients with uneventful healed fractures: 21.4° and 13.8°, respectively (*p* = 0.03). The failure rate was 3.2% for Garden type I and II FNF with a posterior tilt < 20° and 12.5% if the preoperative posterior tilt was ≥ 20°. A posterior tilt of ≥ 20° was associated with an odds ratio of 4.24 (95% CI 1.09–16.83; *p* = 0.04).

**Conclusion:**

Garden type I and II FNFs with a significant preoperative posterior tilt (≥ 20°) seem to behave like unstable fractures and have a four times higher risk of failure. Preoperative posterior tilt ≥ 20° of the femoral head should be considered as a significant predictor for failure of treatment in Garden type I and II FNFs treated with the DLBP.

## Introduction

The Garden classification is most commonly used to describe displacement of femoral neck fractures (FNF) [1]. Garden types I and II are relatively undisplaced or stable FNFs, whereas Garden types III and IV are displaced or unstable fractures. This classification is based solely on the review of anteroposterior (AP) radiographs [2]. The preoperative tilt of the femoral head in the anterior and posterior directions is not included in this classification.

A posterior tilt may be of consequence for the stability of the fracture. Most of the studies describing the clinical relevance of a posterior tilt show that it influences the outcome of treatment, with osteosynthesis of undisplaced FNFs [3–9]. However, the correlation between posterior tilt and treatment failure is not always clear and undisputed [10, 11]. Several researchers have used 20° as the cut-off point above which posterior tilt is assumed to be relevant to the clinical outcome; however, this value is only founded by a few authors [4, 7, 9]. Most fixations used in these studies concerned cannulated screws or sliding hip screws. Little is known about the influence of posterior tilt when other implants are used.

The dynamic locking blade plate (DLBP) is a relatively new implant with demonstrated increased fracture-implant construct stability. It has been used for fixation of displaced and undisplaced FNFs since 2010 [12–14].

In this study, we aimed to determine the correlation between the preoperative posterior tilt of the femoral head and the treatment failure rate in patients with a Garden type I and II FNF treated with the DLBP.

## Patients and methods

### Set up

Five hospitals in the Netherlands that all used the dynamic locking blade plate (DLBP, Baat Medical, Hengelo, Netherlands) as a standard of care for fixation of FNFs prospectively collected data on patients with hip fractures who had been treated with the DLBP. Patients in these hospitals were treated according to “the Dutch guideline for the treatment of proximal femoral fractures” with the use of the DLBP for internal fixation for the fracture [15]. These data were retrospectively analyzed.

The measurements and analysis were in line with earlier performed studies within a research program that had been assessed by the Medical Research Ethics Committee (MREC). The MREC concluded that the studies do not meet the criteria to be evaluated by a MREC and can consequently be performed without official MREC approval.

### Patients

All patients with a FNF who were treated between January 8, 2010 and January 1, 2015 with the dynamic locking blade plate were identified. Patients with a Garden I or II FNF were included for further analysis. Patients with pathological fractures, concomitant fractures of the lower extremity, symptomatic arthritis, local infection or inflammation, open fractures, morbid obesity classified as a body mass index of ≥ 35 kg/m^2^, and a mental or neurological disorder that could impair successful healing of the fracture were excluded.

### Data acquisition and outcome

The data on the following were collected by the treating trauma surgeons: sex, age, 4-grade Garden classification, time to surgery, operation time, pre-reduction posterior tilt, quality of reduction (an angle between 155° and 180° on the anteroposterior (AP) and lateral X-rays was considered to be a good reduction) [16] and tip-apex distance (TAD) [17]. The mean follow-up was 1 year and the range for follow-up was also 1 year. Appointments for follow-up and postoperative X-rays were made directly postoperatively, at 6 weeks, 3 months, 6 months, and 1 year or until the primary endpoint was reached.

The primary outcome was failed treatment in terms of avascular necrosis of the femoral head (AVN), non-union, implant cut-out, with the need for revision surgery. Non-union was defined as persistent pain in the hip or inability to bear weight in combination with a visible fracture line or absence of cortical bridging or bridging trabeculae over the fracture site on the AP and lateral radiographs at least 4 months postoperative, which led to revision surgery.

Avascular necrosis was defined as persistent pain in the hip or inability to bear weight in combination with at least stage 2 AVN according to the Steinberg classification [18]. The Steinberg classification uses the AP and lateral radiographs to assess AVN wherein stage 2 is defined as an abnormal radiograph which shows “cystic” and sclerotic changes in the femoral head. Cut-out of the implant was defined as breakage or cut-out of the blade, plate, or screws; inadequate expansion/malfunction of the anchors; or any malfunction of the implant that led to revision surgery.

### Fracture classification and posterior tilt measurement

**Fig. 1:**
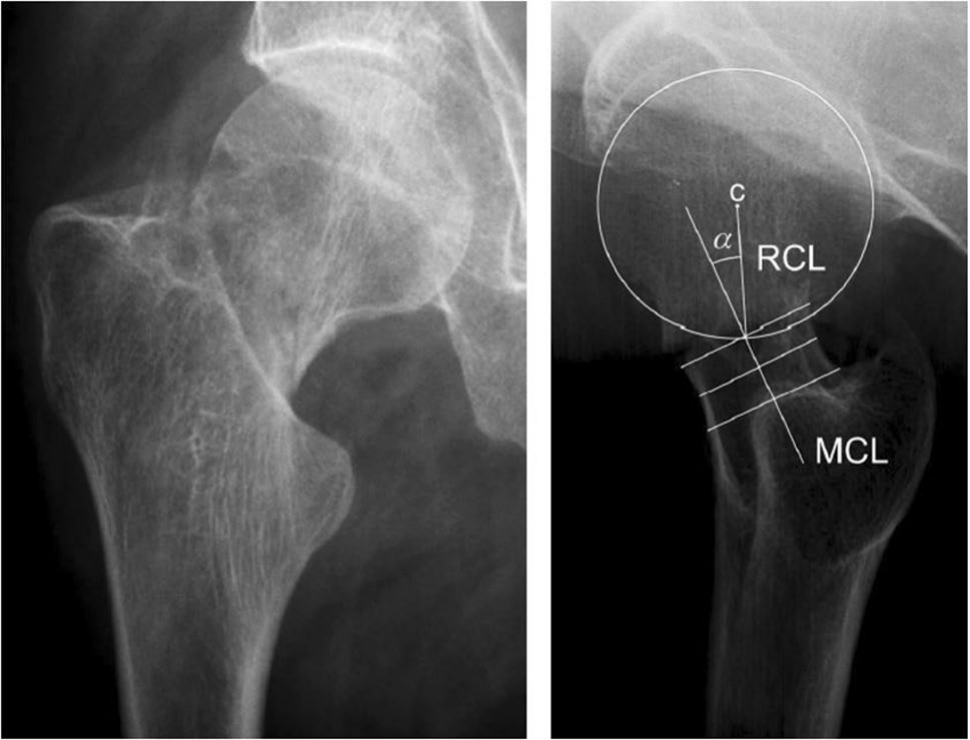
The posterior tilt measurement (PTM) according to palm [4] is the angle (α) between the mid-collum line (MCL) and the radius collum line (RCL)

For the X-rays, patients were positioned in supine position, the contralateral leg positioned with both hip and knee in 90° flexion. If needed the foot was supported. The injured leg was positioned in its natural position with slight exorotation of the foot. The X-ray generator was positioned horizontally perpendicular to the detector, and the detector was positioned parallel to the femoral neck at the lateral side of the pelvis. Fractures were classified as Garden type I and II or Garden type III and IV by the treating surgeon and re-classified by the first author (JHK) to avoid single observer bias. In case of any discrepancy in the classification or measurement between the first author and the treating surgeon, the case was reviewed and discussed with the second author (ADPW). In all Garden type I and II fractures, the preoperative posterior tilt on the lateral view was measured by the first author (JHK) according to the posterior tilt measurement (PTM) [4]. The degree of posterior tilt of the femoral head was determined by the angle between two lines, the mid-collum line (MCL) and the radius collum line (RCL) (Fig. 1). The middle of the femoral neck was determined by drawing three perpendicular lines across the narrowest part of the collum, with 5 mm between each line. The RCL was drawn from the middle of the femoral head to the intersection of the MCL and the caput circle [4].

### Surgical treatment

**Fig. 2:**
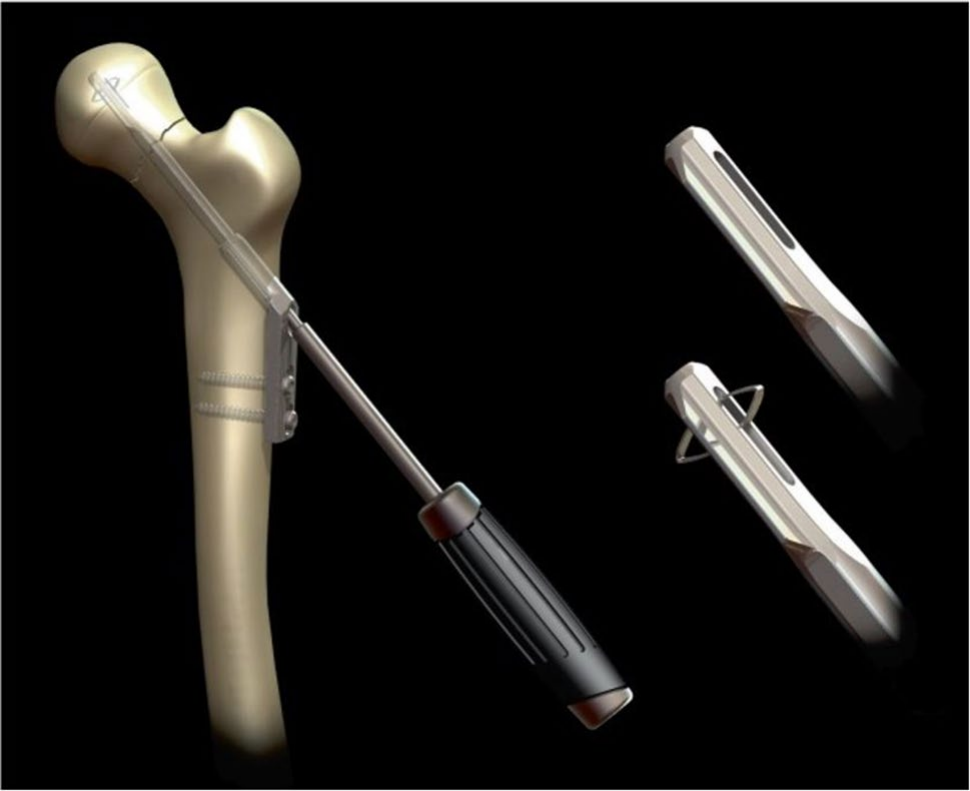
Dynamic locking blade plate

Reduction was performed using a closed technique for all the fractures. The FNFs of all included patients were fixated with the DLBP. The DLBP is a barreled side-plate combined with a cannulated locking blade (Fig. 2) [13, 14]. Perioperative care was given according to the local hospital protocols, including pre-operative antibiotic prophylaxis, direct full-weight-bearing of the operated hip after surgery according to patients’ pain perception and functional capacities, and antithrombotic prophylaxis.

### Statistics

Statistical analysis was performed with SPSS v. 2 software (IBM Corp., Armonk, New York) for Windows 7 (Microsoft, Redmond, Washington). Baseline characteristics are displayed as mean with SD or median range for continuous variables. Categorical variables are displayed as numbers with corresponding percentages. Differences in baseline characteristics between healed and failed fractures were tested with an independent *t* test or Mann–Whitney *U* test depending on the distribution of the continuous data. For categorical data, the Chi-square test was used.

Several cut-off points for posterior tilt that are highly associated with failure are described in literature [4, 5, 7]. To find a cut-off point with the highest clinical relevance, the data were also classified in groups with a posterior tilt either less than or greater than/equal to 10°, 15°, 20°, and 25°. To test the association between dichotomized posterior tilt and failure, a univariate logistic regression analysis was performed. Potential confounders from Table 1 that were associated with posterior tilt and with failure (*p* value < 0.15) were taken into account in the multivariate logistic regression analyses. *P* values less than or equal to 0.05 were considered to be statistically significant.

**Table 1:** Demographics and outcome measurements for healed versus failed fractures

	Healed FNF *n*=155	Failed FNF treatment *n*=9	*p* value
Mean age in years (SD)	68.3 (14.2)	73.7 (9.3)	0.13
Female, *n* (%)	94 (60.6)	7 (77.8)	0.48
Mean TAD in millimeters (SD)	21.0 (6.4)	24.2 (7.7)	0.15
Malreduction, *n* (%)	10 (6.5)	2 (22.2)	0.13
Mean PTM in degrees (SD)	13.8 (10.4)	21.4 (11.1)	0.03
Operation time in minutes (SD)	42 (18.4)	46 (19.1)	0.49

*FNF* femoral neck fracture, *SD* standard deviation, *TAD* tip-apex distance, *PTM* posterior tilt measurement.

## Results

### Patients

**Fig. 3:**
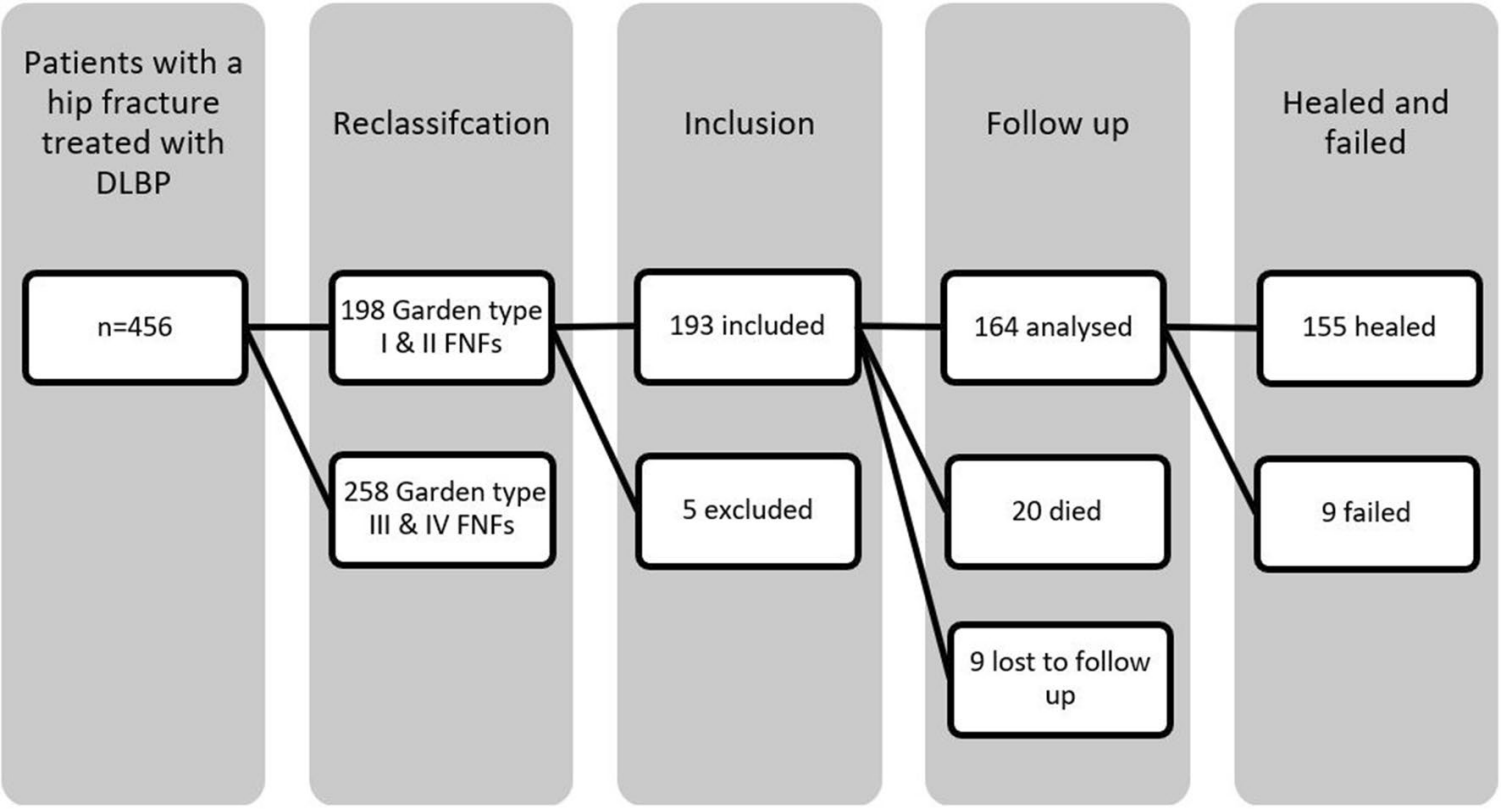
Flowchart of patient inclusion and follow-up. DLBP, dynamic locking blade plate; FNF, femoral neck fracture

There were 456 patients with a hip fracture who were treated with DLBP. The flowchart is shown in Fig. 3. In 46 of the 456 cases there was discrepancy between the initial and second classification. Consensus was reached in all of the cases after review by a third party [ADPW].

Of the 456 patients, 198 had Garden type I and II and 258 Garden type III and IV FNFs. Two patients with a Garden type I and II FNF had concomitant fractures of the lower extremity, and three patients had a mental and/or neurologic disorder. In this study, 193 patients were included for analysis. Twenty of these patients died during follow-up. None of these patients died as a result of the operation or a related complication. Eight patients were lost to follow-up (moved abroad or had follow-up in hospitals not participating in this study), and for one patient, no preoperative X-rays could be obtained.

The mean age of the 164 patients with a Garden type I and II FNF was 68.5 years (range 35–101) and 61.6% were female. The time (or delay) until operation was registered as either within 24 h or after 24 h. Of the 164 patients, 21% (*n *= 36) were not treated within 24 h. There was no statistical difference between the healed and failed group in terms of delay to surgery.

### Failure of treatment

In total, the treatment failed in nine of the 164 patients (5.5%). These nine patients all needed revision surgery. Three patients had AVN diagnosed on X-ray, two patients had non-union of the fracture, one patient had non-union with clinical suspicion for AVN but not seen on the X-ray, and three patients had cut-out of the implant. In 12 patients, the femoral head had not been properly reduced and posterior tilt persisted. Treatment failed in two of these patients.

### Posterior tilt of the femoral head

Table 1 shows the demographics of the patient groups with healed fractures versus failed fractures. The mean posterior tilt was 14.2° (SD = 10.6). Patients with failed fracture treatment had more posterior tilt on the initial radiograph than the patients with healed fractures, 21.4° versus 13.8° (*p *= 0.03). Failure of treatment tended to be associated with an older age, a greater TAD, and malreduction, but the associations were not statistically significant.

After classification into groups with the posterior tilt of the femoral head less than or greater than/equal to 10°, 15°, 20°, and 25°, no differences were found between the patients with healed and failed fractures in the groups 10°, 15°, and 25° (Table 2). If posterior tilt angles were divided into < 20°and ≥ 20° groups, we did find statistically significant differences between these groups, as shown in Table 3 (*p* = 0.04).

**Table 2:** Odds ratios of dichotomized PTM into groups < and ≥ than 10°, 15°, 20° and 25° posterior tilt of the femoral head related to healed and failed fractures

Group	Odds ratio	*p* value	95% confidence interval
PTM > 10°	5.9	0.08	0.72–48.60
PTM > 15°	3.5	0.08	0.85–14.69
PTM > 20°	4.2	0.04	1.09–16.83
PTM > 25°	3.8	0.09	0.88–16.56

*PTM* posterior tilt measurement

**Table 3:** Crosstab of dichotomized PTM into < 20° and ≥ 20° posterior tilt of the femoral head related to healed and failed fractures

	Healed FNF	Failed FNF treatment	Total
PTM ≥ 20°	35 (87.5%)	5 (12.5%)	40
PTM < 20°	120 (96.8%)	4 (3.2%)	124
Total	155 (94.5%)	9 (5.5%)	164

Odds ratio = 4.24 (95% CI 1.09–16.83; *p *= 0.04)

*PTM* posterior tilt measurement, *FNF* femoral neck fracture

Posterior tilt of ≥ 20° was associated with an OR of 4.24 (95% CI 1.09–16.83; *p* = 0.04). No differences were found between patients with a posterior tilt < 20° and ≥ 20° in terms of gender (*p* = 0.20), TAD (*p* = 0.37), and malreduction (*p *= 0.73). Patients with a posterior tilt ≥ 20° were on average 5.4 years younger (mean < 20° = 69.9, mean ≥ 20° = 64.5) than patients with a posterior tilt < 20° (*p* = 0.033). If we corrected the odds ratio (OR) of the posterior tilt angle ≥ 20° for age, the OR increased to 5.36 (95% CI 1.30–22.11; *p* = 0.02).

## Discussion

This study aimed to determine the correlation between the preoperative posterior tilt of the femoral head on the initial radiograph and the treatment failure rate in patients with a Garden type I and II FNF after fixation with the dynamic locking blade plate (DLBP). A larger posterior tilt was associated with treatment failure. A posterior tilt of ≥ 20° was associated with a four times higher failure rate.

It is well known that the displacement of the femoral head caused by a FNF potentially compromises the vascularization of the femoral head. Most of the blood supply to the femoral head originates from the fragile lateral epiphyseal arteries that originate from the retinacular or subsynovial arteries that form an intracapsular ring in the hip joint [19]. After displacement of the femoral head, these vessels can be torn or kinked and this may result in an impaired blood flow, which can be devastating for the vitality of the femoral head. This impaired blood flow may also occur in FNFs with varus angulation on the AP X-ray and posterior tilt on the lateral X-ray. Posterior tilt of the femoral head could result from posterior comminution of the femoral neck (Fig. 1), and posterior comminution is associated with non-union and AVN [20, 21]. After reduction of the fracture, a gap remains in the posterior cortex, which results in an unstable posterior border despite optimal reduction. In case of insufficient bone stock or inadequate fixation, this posterior instability may lead to an early collapse of the femoral head and treatment failure. This underpins the importance of optimal visualization of the fracture for classification purposes. Subsequently, the lateral X-ray has to be included in the assessment of the fracture and the fracture classification. An FNF with posterior tilt needs to be recognized preoperatively and has to be treated as a fracture that is presumably unstable, even when the AP classification shows a Garden type 1 or 2 fracture.

Several researchers have published articles on the influence of posterior tilt. Alho et al. [10] were the first to describe the correlation between posterior tilt and failure of treatment in 1992. Their study, however, included only 13 undisplaced FNFs, which did not allow for any definitive conclusions. In a retrospective analysis of 375 undisplaced FNFs, Conn and Parker demonstrated an association between a larger posterior tilt and non-union but no association between posterior tilt and AVN [3]. In 2013 Clement et al. concluded that posterior tilt was a significant predictor of fixation failure [5]. Clement et al. defined a posterior tilt as a lateral Garden angle (LGA) of < 170°. No substantiation was given for the number of degrees. Following these findings in literature, we did an additional analysis in which we categorized our data into a posterior tilt < 10° and ≥ 10°, but this did not demonstrate any significant correlation between posterior tilt and failure in our data. Clement et al. used the LGA to measure posterior tilt of the femoral head. We believe that the LGA is a reliable method to measure; however, it is inferior to the PTM of Palm et al., which has a better interobserver reliability [22].

Palm et al. showed in a retrospective analysis that a posterior tilt of ≥ 20° is a significant predicator for failure of non-displaced FNF treated with cannulated screws [4]. Dolatowski et al. also found an increased risk of fixation failure with a hazard ratio (HR) of 2.4 (95% CI 1.1–5.4); *p* = 0.03) for posterior tilt ≥ 20° after dichotomization of the angle [7]. In 2019, Dolatowski et al. did another analysis on the influence of posterior tilt. In 111 patients treated with internal fixation, an increased HR of 2.2 for healing-related complications was found (95% CI 1.2–4.0); *p* = 0.008) in patients with a posterior tilt of ≥ 20° [23]. In 2019 Sjöholm et al. did a retrospective analysis of 417 patients with an undisplaced FNF and found an HR of 1.5 (95% CI 1.2–2.0) for posterior tilt ≥ 20° [9]. They also found an increased risk for failure for anterior tilt of more than 10° (HR 2.9; 95% CI 1.4–6.3). We did not analyze anterior tilt in this cohort because the anterior tilt was not measured in any of the patients.

Sjöholm et al. analyzed the influence of 30° and 40° posterior tilt [9]. Posterior tilt of 30° and 40° show an HR of 3.3 (95% CI 1.7–6.4) and 7.0 (95% CI 2.4–21), respectively. We could not find a significant difference in odds ratios for higher or smaller cut-off points of posterior tilt. This was probably because of the small number of failures in our study. Okike et al. did a secondary analysis of 555 patients with a undisplaced FNF treated with internal fixation which showed an HR of 2.2 (95% CI 1.2–4.0; *p* = 0.008) if posterior tilt was higher than 20° [8]. In 2013, Lapidus et al. performed the only large study that contradicted the influence of posterior tilt on failure in Garden type I and II FNF treatment [11]. They measured the posterior tilt in 382 Garden type I and II FNFs. Their results conflict with current literature and the results in our study.

Despite varying results, different outcome parameters, different implants, and heterogeneity of study populations, posterior tilt seems to be a predisposing factor for failure in treatment with internal fixation in Garden type I and II FNF. This is confirmed in a recently published systematic review and meta-analysis by Nielsen et al. [24].

The indications for fracture fixation with a DLBP were the same as those for other internal fixation devices such as the dynamic hip screw or cannulated screws, and the hospitals followed the recommendations stated in the Dutch guideline for treatment of proximal femoral fractures [15]. In this national guideline and the current guideline for treatment of proximal femoral fractures [25], the lateral X-ray is not included in assessing the characteristics of the fracture and determining the stability and angulation of the FNF.

To analyze the position of the blade of the DLBP in the femoral head we measured the TAD. In our study, an increased TAD was not associated with a higher failure rate. We also tested the TAD as a potential confounder. The odds ratio of ≥ 20° posterior tilt did not significantly change when corrected for the TAD (*p* = 0.37). A possible explanation for the limited influence of the head load carrier position may be the high stability of the DLBP blade compared to femoral head screw of other implants used for fixating FNF [26].

**Fig. 4:**
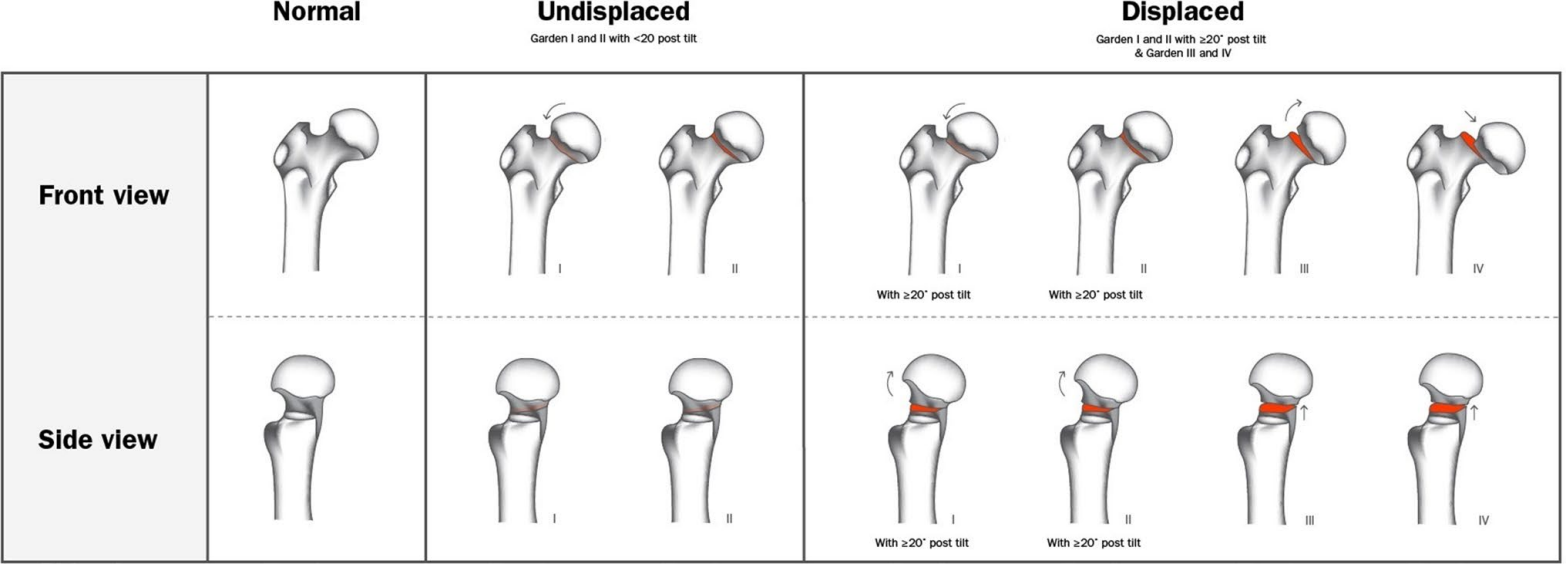
Proposed modified Garden classification of undisplaced and displaced femoral neck fractures, taking into account the amount of initial posterior tilt (displacement)

Our results show failure rates of 3.2% for the Garden type I and II FNF with a posterior tilt < 20° and 12.5% if the posterior tilt is ≥ 20°. These numbers are similar to the failure rates that we found for displaced (Garden III and IV) FNFs in patients age 60 and younger treated with the DLBP [14]. It seems that “stable,” undisplaced, Garden type I and II FNF with significant posterior tilt (≥ 20°) behave like unstable fractures. Therefore, despite the use of the original Garden classification for decades, we suggest a modified Garden classification wherein Garden type I and II FNFs with a posterior tilt of < 20° are classified as undisplaced fractures and Garden type I and II with posterior tilt of ≥ 20° and type III and IV as displaced fractures (Fig. 4). Palm et al. already included posterior tilt into a new algorithm in 2012 [27], but they also incorporated vertical fractures into the algorithm. We think that vertical fractures according to the Pauwels classification should not be incorporated in a treatment algorithm, since the reliability of the Pauwels classification is limited and has low predictive value with regard to outcome [28, 29]. However, we do believe the proposed modified Garden classification could influence the treatment strategy that we use today. We expect that the treatment strategy for FNFs in elderly patients with a Garden type I and II fracture will shift from fracture fixation to hip replacement when the posterior tilt of the femoral head is greater than 20°.

A strength of this study is the fact that there was no missing data of patients treated with the DLBP because of the prospective documentation of the outcome parameters. However, this study also has some limitations. The most important one is the small number of patients with failed treatment. A consequence was that we, due to the small numbers after dichotomization, could not find significant differences in odds ratios for higher or smaller cut-off points of posterior tilt. Therefore, we could not assess if posterior tilt < 20° also significantly influences the treatment. Another limitation was posed by the inability to perform a multivariate analysis to correct for the case mix of all patient-related parameters. As a result, we could not determine the influence of the specific variables and their relevance, nor could we confirm the association found after univariable analysis of posterior tilt as a predictor of treatment failure. However, after correction for age, one of the probably dominant variables of influence, the OR for failure related to posterior tilt, increased.

## Conclusion

The results of this study show that posterior tilt of 20° or more was associated with a four times higher failure rate in Garden type I and II FNFs treated with the DLBP. It seems that “stable” undisplaced, Garden type I and II FNF with a significant posterior tilt (≥ 20°) in fact behave like unstable fractures. Therefore, a preoperative posterior tilt ≥ 20° of the femoral head should be considered as a significant predictor for failure of treatment in FNF treated with the DLBP. An adapted Garden classification that includes the posterior tilt for Garden type I and II fractures may prove helpful in future choices of treatment and subsequent prevention of failure of treatment with osteosynthesis.

## Data Availability

Data will be available upon request.

## Code availability

Statistical analysis was performed with SPSS v. 2 software (IBM Corp., Armonk, New York) for Windows 7 (Microsoft, Redmond, Washington).
